# Recent Technological Upgrades to the SHYPROM IoT-Based System for Monitoring Soil Water Status

**DOI:** 10.3390/s25164934

**Published:** 2025-08-09

**Authors:** Alessandro Comegna, Shawkat Basel Mostafa Hassan, Antonio Coppola

**Affiliations:** 1 Department of Agricultural Forestry Food and Environmental Sciences (DAFE), University of Basilicata, 85100 Potenza, Italy; shawkat.hassan@unibas.it; 2 Department of Chemical and Geological Sciences, University of Cagliari, 09042 Cagliari, Italy; antonio.coppola@unica.it

**Keywords:** IoT system, capacitive-based sensor low-cost systems, soil sensors, soil water content, matric potential, hydraulic conductivity

## Abstract

**Highlights:**

A cost-effective IoT device was developed to support hydrological monitoring tasks.

The device performed consistently well across soils with varying textural compositions.

Controlled laboratory tests confirmed the system’s precision in estimating key soil parameters. This system proves effective for continuous real-time monitoring in both precision agriculture and environmental research.

**What are the main findings?**

- Development of a low-cost hydrological monitoring system.
- Consistent and reliable performance.

**What is the implication of the main finding?**

- Possibility to build the device on one’s own.
- It makes the IoT monitoring system suitable for monitoring soil water status with acceptable accuracy.

**Abstract:**

Effective water resource management plays a crucial role in achieving sustainability in agriculture, hydrology, and environmental protection, particularly under growing water scarcity and climate-related challenges. Soil moisture (θ), matric potential (*h*), and hydraulic conductivity (*K*) are critical parameters influencing water availability for crops and regulating hydrological, environmental, and ecological processes. To address the need for accurate, real-time soil monitoring in both laboratory and open-field conditions, we proposed an innovative IoT-based monitoring system called SHYPROM (Soil HYdraulic PROperties Meter), designed for the simultaneous estimation of parameters θ, *h*, and *K* at different soil depths. The system integrates capacitive soil moisture and matric potential sensors with wireless communication modules and a cloud-based data processing platform, providing continuous, high-resolution measurements. SHYPROM is intended for use in both environmental and agricultural contexts, where it can support precision irrigation management, optimize water resource allocation, and contribute to hydrological and environmental monitoring. This study presents recent technological upgrades to the proposed monitoring system. To improve the accuracy and robustness of θ estimates, the capacitive module was enhanced with an integrated oscillator circuit operating at 60 MHz, an upgrade from the previous version, which operated at 600 kHz. The new system was tested (i.e., calibrated and validated) through a series of laboratory experiments on soils with varying textures, demonstrating its improved ability to capture dynamic soil moisture changes with greater accuracy compared to the earlier SHYPROM version. During calibration and validation tests, soil water content data were collected across a θ range from 0 to 0.40 cm^3^/cm^3^. These measurements were compared to reference θ values obtained using the thermo-gravimetric method. The results show that the proposed monitoring system can be used to obtain predictions of θ values with acceptable accuracy (*R*^2^ values range between 0.91 and 0.96). To further validate the performance of the upgraded SHYPROM system, evaporation experiments were also conducted, and the θ(*h*) and *K*(θ) relationships were determined among soils. Retention and conductivity data were fitted using the van Genuchten and van Genuchten–Mualem models, respectively, confirming that the device accurately captures the temporal evolution of soil water status (*R*^2^ values range from 0.97 to 0.99).

## 1. Introduction

Accurate monitoring of soil moisture content, matric potential, and hydraulic conductivity is essential for understanding soil–water dynamics, optimizing irrigation practices, and promoting the sustainable use of water resources. However, traditional methods for estimating soil hydraulic properties often have limitations in terms of temporal and spatial resolution, labor requirements, and real-time data acquisition [1,2,3,4,5,6,7].

Recent advances in Internet of Things (IoT) technology have paved the way for developing innovative soil monitoring systems, enabling continuous, high-resolution, and remote measurements. These systems typically comprise a network of low-cost, wireless sensors capable of measuring key soil parameters at multiple depths within the soil profile. Sensor-generated data can be transmitted wirelessly to a data acquisition unit, often leveraging cloud computing platforms for real-time analysis and decision-making [8].

In addition to their agricultural applications, IoT-based monitoring systems are increasingly being recognized for their potential in hydrological and environmental monitoring. These systems can also integrate additional environmental sensors, such as those for temperature, *pH*, and electrical conductivity, offering a more comprehensive assessment of soil and environmental conditions [9,10].

The application of IoT technologies in hydrological and environmental monitoring is still evolving, with several challenges yet to be addressed. These include sensor calibration and maintaining data accuracy under variable environmental conditions, as well as ensuring long-term system reliability, particularly in remote or harsh environments [11,12,13,14,15]. Furthermore, integrating IoT-based monitoring systems with existing hydrological and environmental models and decision support frameworks remains a complex task, requiring the development of user-friendly interfaces and robust analytical tools capable of processing large volumes of data [16].

The SHYPROM IoT-based monitoring system represents an innovative solution for real-time monitoring of soil water status, providing reliable and precise data on soil hydraulic parameters. This system leverages low-power wireless sensors capable of long-term deployment, offering a cost-effective approach for large-scale environmental monitoring. It allows for the implementation of a monitoring network composed of multiple SHYPROM devices, in accordance with standard open-field protocols, to ensure adequate coverage of the research areas.

In the study by [1], the authors primarily focused on the conceptual framework and technological features of the proposed monitoring system, as well as on the performance of the SHYPROM device, which was validated through several laboratory tests. As highlighted in their conclusions, the main limitations of that research concerned the soil database used for sensor calibration and validation and the low operating frequency (600 kHz) of the capacitive module integrated into the SHYPROM system.

In general, capacitive-based sensors operate at relatively low frequencies. For this reason, their measurements are influenced by factors such as soil temperature, texture, and salinity [17,18,19,20,21,22,23]. However, extensive research has demonstrated that these influences are significantly reduced when sensors are used in soils with temperatures between 15 and 30 °C and with soil solution electrical conductivity (EC_w_) below 10 dS/m [24].

As the measurement frequency increases, typically around 50 MHz or higher, the effects of temperature and salinity become less pronounced, even beyond the aforementioned thresholds [25]. Nevertheless, the operating frequency of capacitive systems generally cannot exceed 100 MHz. Indeed, studies by [26,27] showed that heavy-textured soils, which contain moderate-to-high amounts of clay minerals, exhibit dielectric dispersion at frequencies above 100 MHz. This phenomenon significantly limits the accuracy of water content determination in such soils [28].

To address these technical limitations, the present research introduces recent technological upgrades to SHYPROM’s capacitive sensor module, which now operates at a frequency of 60 MHz. This frequency represents a suitable compromise between measurement accuracy and the previously discussed limitations.

To evaluate this upgraded version of SHYPROM (now referred to as SHYPROM60), several laboratory tests were carried out using three soils with different textures. For sensor calibration, the estimated θ values obtained with the new capacitive module were compared, as a reference, to measurements acquired through the thermogravimetric method. Furthermore, to assess the performance of the SHYPROM60 device, including the functionality of the source code (i.e., the firmware) controlling the hardware, evaporation experiments were conducted on the selected soils. During these experiments, the θ, *h*, and *K* values were measured, and the θ(*h*) and *K*(θ) relationships were determined.

## 2. Materials and Methods

### 2.1. Hardware Description

The SHYPROM60 monitoring system essentially consists of (i) an ESP32 SIM800L microcontroller board (LilyGO, Shenzhen, China), (ii) a pair of porous tensiometer cups connected to two electronic pressure transducers (MPX5100DP model, Freescale Semiconductor, which works in the 0 to 100 kPa differential pressure range) for soil water matric potential measurement, (iii) three pairs of stainless steel tubular electrodes for soil moisture estimation, and (iv) a temperature sensor (DS18B20, waterproof version).

**Figure 1:**
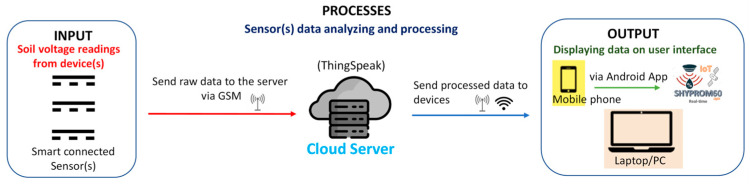
Conceptual framework of the IoT-based sensor platform (adapted from [1]).

Data collected from the integrated sensors can be transmitted wirelessly to a cloud server at fixed time intervals. The stored data can then be visualized on a laptop or directly on a mobile phone via a custom Android-based application (called *SHYPROM60_PRO*), which is provided along with the SHYPROM60 firmware (*SHYPROM60.ino*) in the Supplementary Material. Figure 1 illustrates the conceptual framework of the IoT-based monitoring system (see also [1] for further details).

For soil water content measurements, SHYPROM60 consists of three pairs of electrodes (3 cm high, 2 mm thick, and 2.5 cm in diameter) that operate as capacitors. These electrodes are positioned at different heights on two plexiglass tubes (2 cm in diameter), forming three pairs of electrodes. The plexiglass tubes also serve as tensiometers. Each tensiometer unit comprises a plexiglass column fitted with a porous ceramic cup characterized by a 150 kPa air entry value. The top side of the plexiglass tube is equipped with a pressure transducer glued onto a silicone stopper, which seals the tensiometer.

Additionally, to allow for the wiring of the electrical part of the capacitive module, the part of the tube between two electrodes was covered with a series of PLA (i.e., Polylactic Acid fiber) cylinders (obtained via 3D printing). As recalled in previous sections, the capacitive module of SHYPROM60 has an oscillator operating at 60 MHz (i.e., it produces a 60 MHz square wave). This operating frequency was obtained by integrating the electrical circuit of the monitoring system with a Pierce-type oscillator circuit that works with a quartz crystal oscillator operating at a 3rd overtone frequency. To be operational, this circuit, in addition to the crystal, requires a series of resistors, capacitors, and inductors as well as a digital inverter, which allow the crystal, traversed by electric current, to shift from the fundamental frequency to the overtone frequency (see Appendix A).

**Figure 2:**
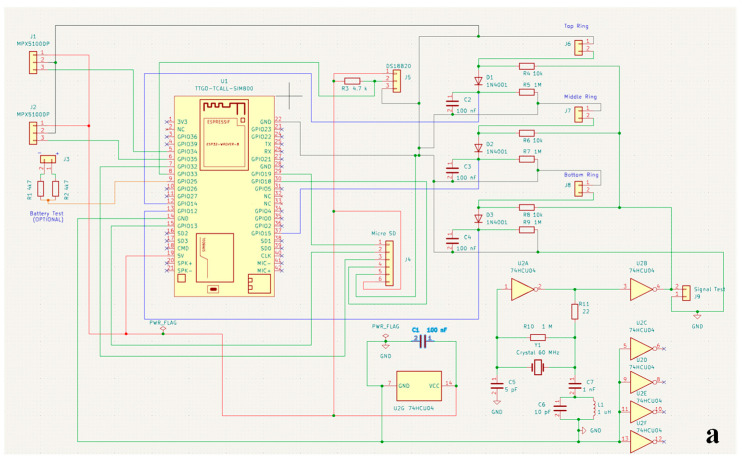

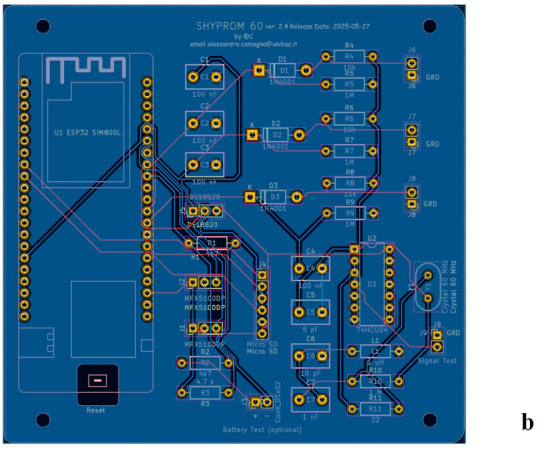
(**a**) Electric circuit diagram of SHYPROM60, and (**b**) the two-layer printed circuit board (PCB) generated by KiCad software.

Figure 2a,b depict the schematic diagram and the dual-layer PCB layout of the SHYPROM60 system, developed through the KiCad software (vers 8.0 https://www.kicad.org/ accessed on 1 January 2025).

Comprehensive documentation, including the firmware managing SHYPROM60′s, hardware functionalities, and details on production costs, is available in Appendix B.

**Figure 3:**
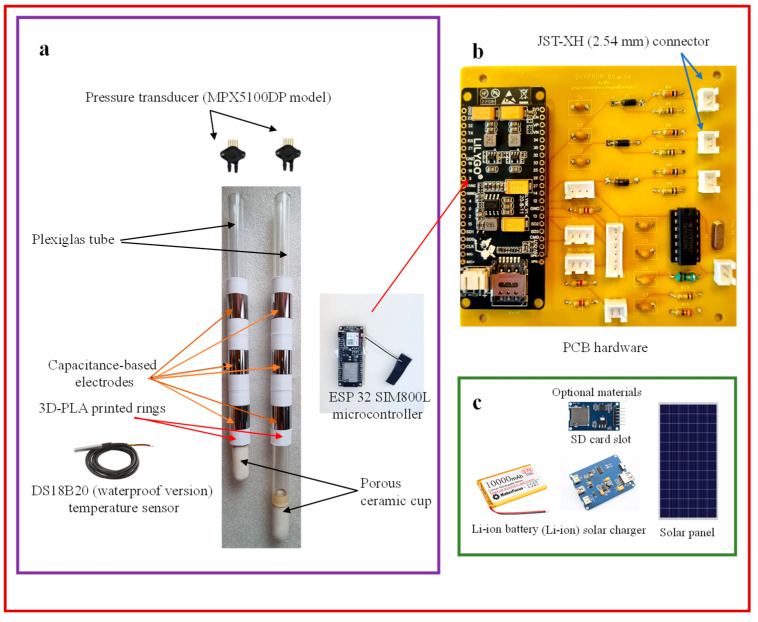
SHYPROM60 hardware: (**a**) main components, (**b**) details of the two-layer printed circuit board (PCB), and (**c**) optional materials (adapted from [1]).

The system features an external micro-SD card interface for data logging purposes. Power is supplied by a 3.7 V, 10,000 mAh lithium-ion battery, which is rechargeable through a waterproof solar panel connected to a dedicated solar charging board. Figure 3a–c illustrate the main hardware components, including the dual-layer PCB shown in Figure 3b, which measures 95 mm by 100 mm.

### 2.2. Soil Properties and Experimental Setup

To calibrate and validate the SHYPROM60 device, several laboratory experiments were conducted on repacked soil samples. Three soils of different textures were selected, classified according to the IUSS Working Group WRB [29] as sandy loam (hereinafter referred to as SALO), silty loam (SILO), and sand (SAND). Table 1 presents the main physico-chemical properties of the soils.

Soil texture and soil bulk density (*ρ_b_*) were determined using the methods described by [30,31], respectively; *pH* and organic content (*OC*) were measured using the methods of [32,33]. The water employed in the experiments had an electrical conductivity (EC_w_) of 0.17 dS m^−1^, as determined using a Cyberscan 500 conductivity meter. Each soil property value was obtained as the average of three independent measurements.

Laboratory activities were divided into two distinct experiments, referred to as exp#1 and exp#2, both conducted on three selected soils. Exp#1 was dedicated to calibrating and validating the capacitive module of the SHYPROM60 system, representing the enhanced technological component of the monitoring platform. Regarding the MPX5100DP transducers, these sensors come with their own factory calibration function. In this case, we only verified (via the hanging water column method) that the pressure transducers operated according to the calibration function provided by the manufacturer. Exp#2 refers to laboratory experiments in which the new capabilities of the SHYPROM60 device were tested by monitoring the evaporation process from bare soil.

In [1], the authors demonstrated the capability of the proposed monitoring system, highlighting its ability to simultaneously determine, using the Instantaneous Profile Method (IPM, [34,35]), the θ, *h*, and *K* values and, hence, the relationships θ(*h*) and *K*(θ). For further details on the IPM and on how to set up its calculation using SHYPROM60, reference is made, for the sake of brevity, to [1].

**Figure 4:**
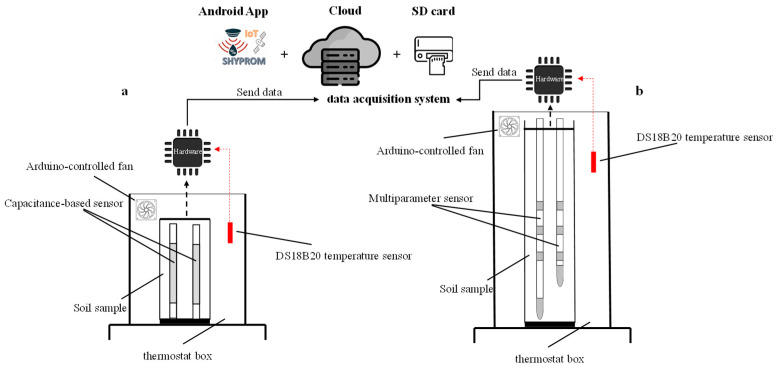
Experimental setup used in (**a**) exp#1 and (**b**) exp#2 (adapted from [1]).

Prior to each experiment, soil samples were dried in an oven at 105 °C and passed through a 2 mm mesh sieve. Figure 4 illustrates the experimental setup of the SHYPROM60 monitoring system used in the laboratory tests.

For the calibration in exp#1, soil samples were repacked using cylindrical PVC containers (9.5 cm in diameter and 10 cm in height). For soil preparation, known amounts of soil and water were mixed together and then kept for 12 h in sealed plastic bags to prevent any evaporation. Once the soil samples were prepared, the capacitive electrodes for θ estimation were inserted vertically into the soil. To prevent water loss during the experiments, the ends of the PVC containers were carefully sealed. The laboratory protocol included a full-factorial set of measurements to determine the volumetric water content across a θ range from 0 to 0.40 for all three soils.

Since capacitive readings are affected by temperature ([36,37], among others), for each experiment (i.e., for a fixed θ value), the soil sample was tested at temperatures ranging from 20 °C to 30 °C in 1 °C steps using a thermostat box.

Throughout exp#1, differences in voltage (*V*) were monitored over the temperature range. At the same time, reference θ values were obtained through the thermo-gravimetric technique, following the approach described by [38]. The acquired data were utilized to establish the sensor calibration curve, linking the measured θ values with the corresponding voltage readings.

For SHYPROM60 validation, a separate dataset was prepared for each soil, following the same approach used during the calibration phase. Following the calibration and validation of the sensors, the complete SHYPROM60 system underwent integrated testing. In exp#2, a series of three evaporation trials were performed at a constant temperature of 25 °C, with time-based measurements of θ and h recorded at three different soil depths. The sensor assemblies were installed within a repacked soil column measuring 400 mm in height and 250 mm in diameter.

### 2.3. Statistical Indices for SHYPROM60 Performance Evaluation

To assess the performance of SHYPROM60′s capacitive sensing module, three statistical indicators were employed: (i) mean absolute percentage error (*MAE*), (ii) mean bias error (*MBE*), and (iii) model efficiency coefficient (*EF*). These indices were calculated using the standard formulations reported in [39,40]:

(1) $$MAE\left(\%\right)=\frac{\left|E_{i}-O_{i}\right|}{N}\cdot 100,$$

(2) $$MBE=\frac{\sum_{i=1}^{N}\left(E_{i}-O_{i}\right)}{N},$$

(3) $$EF=1-\frac{\sum_{i=1}^{N}{\left(E_{i}-O_{i}\right)}^{2}}{\sum_{i=1}^{N}{\left(O_{i}-\bar{O}\right)}^{2}},$$

In the above equations, *O_i_* represents the observed values (measured using the thermo-gravimetric technique), *E_i_* denotes the estimated values (obtained via SHYPROM60 or the TDR100), $\bar{O}$ is the average of the observed dataset, and *N* indicates the total number of observations.

## 3. Results and Discussion

**Figure 5:**
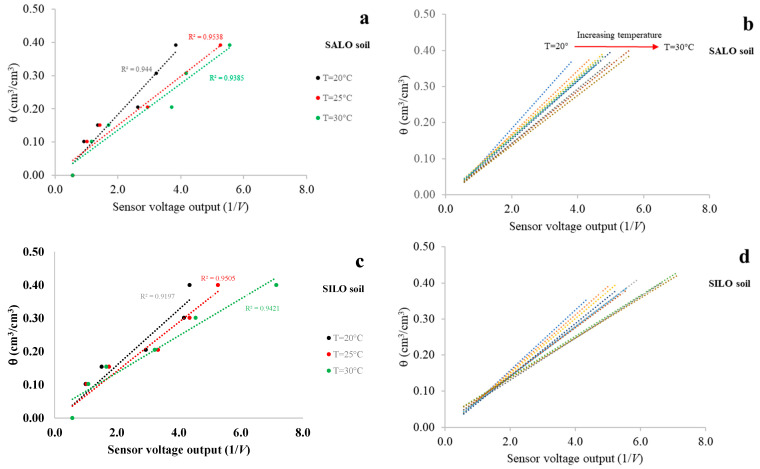

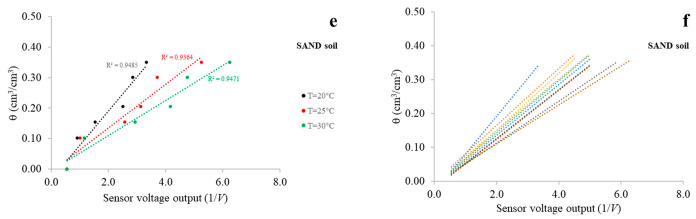
Relationship between θ and 1/*V* for the three investigated soils. Panels (**a**,**c**,**e**) show the results at three selected soil temperatures (20 °C, 25 °C, and 30 °C). Panels (**b**,**d**,**f**) present the corresponding calibration functions across the full temperature range (20–30 °C).

With reference to the three investigated soils, Figure 5 shows the results of the 60 MHz capacitive module calibration. For clarity, Figure 5a,c,e only report the results (with θ values plotted against 1/*V*) at the selected temperatures of 20 °C, 25 °C, and 30 °C. Figure 5b,d,f display the linear calibration functions obtained, with 1 °C steps, across the entire temperature range.

The outcomes of exp#1 highlight the temperature dependence of SHYPROM60. It is worth noting that, for a fixed 1/*V* value, the θ values decrease as the temperature increases. Furthermore, as the soil becomes drier, the differences in θ values across temperatures become less evident (the linear functions tend to converge), since the sensor’s dielectric response is more dependent on soil permittivity [41,42,43,44,45,46]. Additionally, among the soils, in the temperature range of sensor validation, the capacitive-based sensor showed similar trends. This aspect is particularly important as it means that sensor calibration is not soil-dependent, at least with reference to the selected soils.

Table 2 reports the computed *a* and *b* coefficients of the linear regression function and the coefficient of determination (*R*^2^) of the linear calibration functions for the three selected soils. Notably, the strong correlation between θ values and sensor output is confirmed by *R*^2^ values exceeding 0.90 in all cases.

It is worth noting that, unlike the findings reported in [1], where the calibration data were fitted using second-order polynomial functions, the shift in the operating frequency from 600 kHz to 60 MHz enabled the use of linear calibration relationships. This upgrade enhanced the simplicity and robustness of the calibration process.

**Figure 6:**
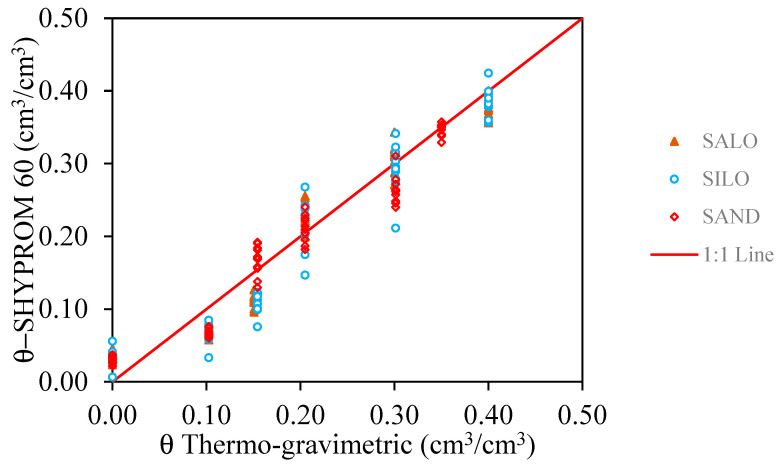
Correlation between the SHYPROM-estimated and measured (thermo-gravimetric method) θ values in the 20–30 °C temperature range, with reference to the three selected soils.

The results of the capacitive sensor validation are shown in Figure 6, where the θ values estimated through the linear calibration functions are compared with the reference θ values obtained via the thermo-gravimetric method. Overall, the estimated values exhibit a strong agreement with the 1:1 line, confirming the accuracy of the sensor. However, at θ = 0.10, all soil types show a slight but consistent underestimation, indicating the sensor’s tendency to undervalue moisture at this content level.

In terms of statistical indices across all soil types and throughout the investigated temperature range, the sensor demonstrated overall reliability in assessing volumetric water content (Table 3). Specifically, *MBE* values ranged between −0.01521 and 0.013107 for SALO, −0.01976 and 0.000762 for SILO, and −0.018688 and 0.00014167 for SAND. In all cases, *MAE* values remained below 3.0. The *EF* values were consistently high, falling within the range of 0.91 to 0.96.

These findings underscore the robustness and precision of the SHYPROM60 device across different soil textures and temperatures and throughout the full moisture range, from saturation to dry conditions.

**Figure 7:**
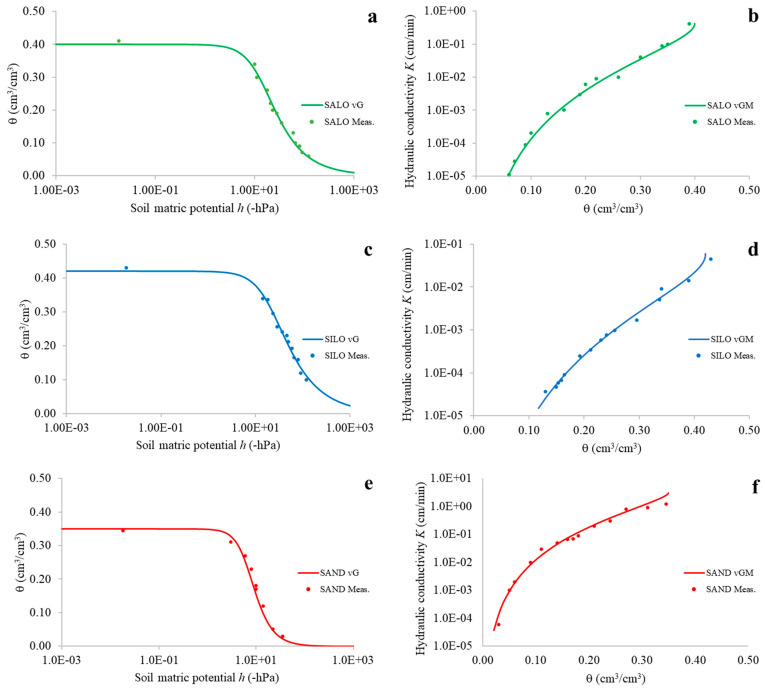
**(a**,**c**,**e**) Experimental soil water retention curves and modeled by the van Genuchten (vG) equation, and (**b**,**d**,**f**) experimental soil hydraulic conductivity function and modeled by the van Genuchten–Mualem (vGM) formula.

With reference to exp#2, an overview of the main outcomes of the SHYPROM60 monitoring system is presented in Figure 7, which shows the experimental and modeled θ(*h*) and *K*(θ) relationships. Modeling was performed using the [47,48] equations, while the *K* values were obtained through the IPM.

Finally, Table 4 reports the van Genuchten (vG) and van Genuchten–Mualem (vGM) model parameters: θ_s_, θ_r_, *α*, *n*, and *K*_0_. These parameters were estimated using the RETC software package [49]. Table 4 also shows the determination coefficients *R*^2^*_vG_* and *R*^2^*_vGM_*. The high values obtained across the different soils indicate that the experimental data estimated using the SHYPROM60 device are of good quality, confirming that SHYPROM60 accurately captures the temporal evolution of soil water status in the three selected soils.

## 4. Conclusions

In the present study, based on a series of full-factorial laboratory experiments, we evaluated the performance of a low-cost IoT-based monitoring system across three soils with different textural characteristics. The research highlights recent technological upgrades to the original SHYPROM monitoring system, most notably the integration of a capacitive module operating at 60 MHz. This enhancement significantly improved the accuracy of volumetric soil water content estimation. SHYPROM60 provides generally more robust water content estimations, also thanks to the possibility of adopting of a linear calibration relationship for the capacitive module, which reduces the number of parameters required for calibration, compared to the third-order polynomial used in the calibration of the previous device version.

Furthermore, coefficients of determination, for the linear equations, consistently exceeded 0.90 regardless of soil type or temperature. Moreover, the updated capacitive sensor demonstrated limited sensitivity to soil type within the range of soils examined. The system’s strong performance was further confirmed by statistical indices (*MBE*, *MAE*, and *EF*), which attested to its high predictive capability and robustness across the full range of soil moisture conditions investigated.

Moreover, validation through evaporation experiments confirmed the reliability of SHYPROM60 in simultaneously determining key soil hydraulic parameters (θ, *h*, and *K*). The resulting water retention and hydraulic conductivity curves were well described by the van Genuchten and van Genuchten–Mualem models, further demonstrating the high quality of the measurements provided by the device.

In addition to its accuracy, the system is also distinguished by its affordability and ease of replication, making it particularly well suited to large-scale applications in precision agriculture and environmental monitoring.

## Figures and Tables

**Table 1:** Basic physico-chemical properties of the selected soils.

Soil ID	Depth (cm)	Soil Texture and Classification (USDA)				*ρ_b_* (g/cm^3^)	*OC* (g/kg)	*pH*
		Texture	Sand (%)	Silt (%)	Clay (%)			
SALO	0–20	sandy loam	57.43	31.95	10.62	1.02	9.5	7.7
SILO	0–20	silty loam	15.7	72.7	11.6	1.02	26.4	8.4
SAND	0–20	sand	98	1.5	0.5	1.02	4.5	7.9

**Table 2:** Estimated regression coefficients *a* (slope) and *b* (intercept) and coefficient of determination (*R*^2^) of the experimental relationships between θ and 1/*V* for the three selected soils and temperature range.

Soil Temperature (°C)	SALO *a*, *b*, *R*^2^	SILO *a*, *b*, *R*^2^	SAND *a*, *b*, *R*^2^
20	0.1028, −0.0221, 0.94	0.0844, −0.0102, 0.92	0.0106, −0.0261, 0.95
21	0.0879, −0.0074, 0.95	0.0794, −0.0044, 0.95	0.0786, 0.0056, 0.91
22	0.0082, −0.0047, 0.96	0.0682, 0.009, 0.96	0.0712, 0.0055, 0.91
23	0.0824, −0.0008, 0.96	0.0762, −0.0017, 0.96	0.0793, −0.0010, 0.94
24	0.0792, 0.0009, 0.93	0.0688, 0.0039, 0.94	0.0709, −0.0095, 0.91
25	0.0733, 0.0049, 0.95	0.074, −0.0067, 0.95	0.0672, −0.0004, 0.92
26	0.0813, −0.048, 0.96	0.057, 0.026, 0.94	0.0721, −0.0029, 0.93
27	0.089, −0.008, 0.96	0.0735, −0.0045, 0.92	0.0687, −0.0124, 0.96
28	0.0726, −0.003, 0.96	0.0666, 0.102, 0.94	0.069, −0.0153, 0.95
29	0.0751, −0.0035, 0.95	0.0583, 0.015, 0.95	0.0587, −0.005, 0.93
30	0.0699, −0.0035, 0.94	0.0561, 0.0238, 0.94	0.0544, 0.0016, 0.94

**Table 3:** Mean bias error (*MBE*), mean absolute percentage error (*MAE*), and model efficiency (*EF*), calculated with respect to the measured and predicted θ values for the three selected soils and temperature conditions.

Soil Temperature (°C)	SALO *MBE*, *MAE*, *EF*	SILO *MBE*, *MAE*, *EF*	SAND *MBE*, *MAE*, *EF*
20	−6.55E-05, 2.74, 0.94	5.86E-04, 3.66, 0.92	−4.09E-03, 2.67, 0.95
21	−1.87E-02, 2.81, 0.93	6.54E-04, 2.51, 0.95	−1.52E-02, 2.82, 0.91
22	−4.80E-03, 2.47, 0.95	−1.73E-02, 2.71, 0.94	−1.50E-02, 2.70, 0.91
23	2.90E-05, 2.01, 0.96	6.01E-04, 2.19, 0.96	−4.15E-03, 2.05, 0.95
24	−1.74E-03, 3.12, 0.93	−7.20E-03, 3.11, 0.93	−4.15E-03, 2.76, 0.92
25	−9.88E-03, 2.39, 0.95	5.71E-04, 2.76, 0.95	−4.26E-03, 2.50, 0.93
26	−1.62E-04, 2.38, 0.96	7.42E-04, 2.98, 0.93	−4.03E-03, 2.36, 0.95
27	6.43E-05, 3.04, 0.93	7.62E-04, 3.25, 0.92	1.31E-02, 2.18, 0.96
28	1.42E-04, 2.44, 0.96	−1.98E-02, 3.61, 0.92	−4.11E-03, 2.62, 0.95
29	−3.71E-05, 2.87, 0.95	−1.30E-02, 2.45, 0.94	−4.15E-03, 2.53, 0.94
30	−2.18E-05, 2.88, 0.94	5.61E-04, 2.62, 0.94	−7.26E-03, 2.48, 0.94
Overall *	**−3.52E-03, 2.65, 0.95**	**−1.05E-02, 2.90, 0.94**	**−5.33E-03, 2.51, 0.94**

* i.e., calculated across the temperature domain.

**Table 4:** van Genuchten (vG) and van Genuchten–Mualem (vGM) model parameters θ_s_, θ_r,_ *α*, *n*, and *K*_0_ and *R*^2^*_vG_* and *R*^2^*_vGM_* values derived from experimental retention and conductivity relationships with reference to the selected soil.

Soil	θ_s_ (cm^3^/cm^3^)	θ_r_ (cm^3^/cm^3^)	*α* (1/cm)	*n*	*K*_0_ (cm/min)	*R* ^2^ *_vG_*	*R* ^2^ *_vGM_*
SILO	0.42	0.0	0.048	1.743	0.006	0.99	0.98
SALO	0.40	0.0	0.075	1.890	0.042	0.98	0.98
SAND	0.35	0.0	0.145	2.680	3.0	0.99	0.97

**Figure A1:**
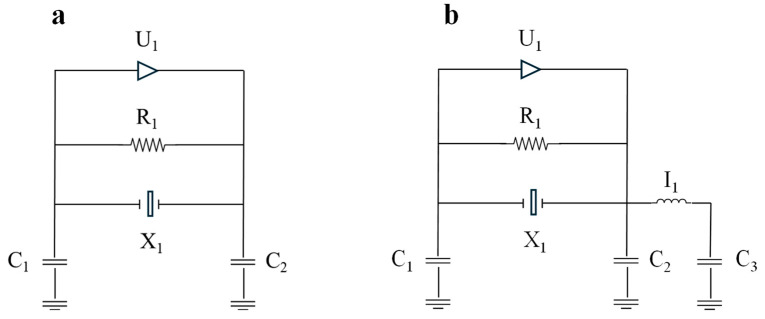
Example of (**a**) a single Pierce circuit working at fundamental frequency, (**b**) Pierce circuit working at overtone frequency.

**Figure A2:**
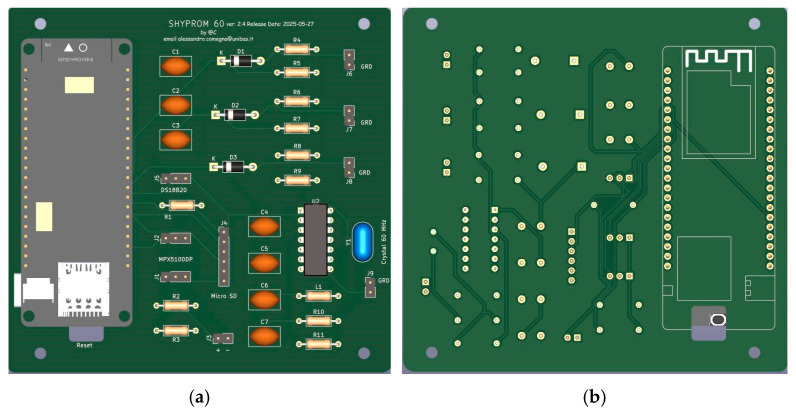
Example of PCB board assembled with all the electronic components: (**a**) front panel, (**b**) back panel.

## Data Availability

The original contributions presented in the study are included in the article/Supplementary Material; further inquiries can be directed to the corresponding author.

## Supplementary Materials

The following supporting information can be downloaded at: https://www.mdpi.com/article/10.3390/s25164934/s1.

## Appendix A. Third Overtone Operation in the Pierce Oscillator Circuit

The Pierce oscillator circuit is a common configuration for use with piezoelectric crystals, known for its simplicity and effectiveness in generating stable signals [50,51,52].

**Operation at the Third Overtone:**

A crystal can oscillate at its fundamental frequency or one of its odd harmonics (overtones), such as the third, fifth, etc. To force the oscillation at the third overtone, the circuit must favor this frequency and suppress the fundamental one.

**Implementation in the Pierce Circuit:**

To achieve oscillation at the third overtone in a Pierce oscillator, an LC resonant circuit (inductor and capacitor) tuned near the crystal’s third harmonic can be introduced. This LC circuit helps suppress the oscillation at the fundamental frequency and promotes the desired third overtone.

For example, adding an inductor in parallel with one of the capacitors in the Pierce circuit can block the fundamental frequency and allow for oscillation at the third overtone.

**Design Considerations:**

- The LC circuit must be designed to present a high impedance at the fundamental frequency, preventing oscillation at this frequency.
- At the same time, the circuit must present adequate impedance at the third harmonic, facilitating oscillation at this frequency.
- Careful selection of inductance and capacitance values is crucial to ensure that the resonant circuit is correctly tuned and functions as intended.

Using the third overtone allows for higher frequencies without requiring crystals with correspondingly high fundamental frequencies, which can be harder to manufacture and less stable. However, designing such circuits requires attention to ensure oscillation occurs at the desired frequency and that unwanted harmonics are effectively suppressed.

In summary, by appropriately modifying the Pierce circuit with the addition of a tuned LC resonant circuit, stable oscillation at the third harmonic of a crystal can be achieved, leveraging the advantages of higher frequencies while maintaining the stability and reliability offered by quartz crystals. Figure A1 shows two examples of a Pierce circuit. The basic device (Figure A1a) is composed of a digital inverter (U_1_), a resistor (R_1_), a quartz crystal (X_1_), and two capacitors (C_1_ and C_2_). Figure A1b shows a modified Pierce circuit working at overtone frequency.

## Appendix B. Supplementary Information

This section outlines the components and resources required for assembling the SHYPROM60 monitoring system. All essential files, such as the firmware, PCB design files, and 3D-printable models, are provided in the Supplementary Material alongside this paper. The structure of the Supplementary Material folder is organized as follows:

1. Board Fabrication Files folder:

(a) The Gerber files (SHYPROM 60 MHz ver 2.4 (two layers).kicad_pcb_gerber.zip);
(b) The Centroid file (SHYPROM 60 MHz ver 2.4 (two layers).kicad_pcb_positions);
(c) The BOM (SHYPROM 60 MHz ver 2.4 (two layers).kicad_pcb_bom).

These files are fundamental for PCB implementation in specialized electronics manufacturing services. Note that the PCB is a two-layer version. This means that printing the PCB is cheaper than the previous versions of SHYPROM.

2. SHYPROM60 Firmware folder:

(a) The Arduino IDE-based source code utilized to program the SHYPROM60 device (SHYPROM60.ino);
(b) Source code for the SHYPROM60_PRO APP (for Android-based devices only: SHYPROM60_PRO.aia).

3. 3D Printing Materials folder:

(a) SHYPROM60_BOX Folder: external waterproof box, .stl files for containing the whole monitoring system;
(b) SHYPROM_RINGS Folder: .stl files for assembling the capacitive electrodes;
(c) How to assemble the SHYPROM60 sensor: a comprehensive user guide for realizing and assembling the SHYPROM60 device.

All 3D-printable components were created using Autodesk’s Fusion 360 software (https://www.autodesk.com).

It is advisable to refer to a specialized electronics manufacturing service that offers PCB production. This option requires the Gerber files, and, in the case of factory assembling the BoM files, for the latter, see Table 2. Figure A2 shows the SHYPROM60 PCB assembled with all the electronic components.

**Details on production costs**

For implementing the electronic part of the SHYPROM monitoring system, two options are possible. The first option is to construct the board by yourself, following the electrical schematic. The estimated cost of fabricating one piece, including materials, is approximately USD 15–20 for the ESP32SIM800L board, USD 50–55 for two MPX5100DP pressure transducers, and around USD 2 for the other required components (listed in Table A1).

For field applications, it is advisable to utilize a specialized electronics manufacturing service capable of producing low-cost printed circuit boards (PCBs). This option requires the Gerber and BoM files. In this case, fabrication costs are around USD 5 for printing ten PCBs, excluding shipping.

Table A1 outlines the electronic components (collectively referred to as the Bill of Materials: BOM) required for the construction of the IoT-based monitoring platform.

**Table A1:** List of electronic components (BoM) required for assembling SHYPROM60.

Footprint Assignment	Designator	Quantity	Footprint Specification (Kikad)	Mounting Type
C1	Capacitor: 100 nF	1	C_Disc_D8.0mm_W5.0mm_P5.00mm	THT *
C2	Capacitor: 100 nF	1	C_Disc_D8.0mm_W5.0mm_P5.00mm	THT
C3	Capacitor: 100 nF	1	C_Disc_D8.0mm_W5.0mm_P5.00mm	THT
C4	Capacitor: 100 nF	1	C_Disc_D8.0mm_W5.0mm_P5.00mm	THT
C5	Capacitor: 5 pF	1	C_Disc_D8.0mm_W5.0mm_P5.00mm	THT
C6	Capacitor: 10 pF	1	C_Disc_D8.0mm_W5.0mm_P5.00mm	THT
C7	Capacitor: 1 nF	1	C_Disc_D8.0mm_W5.0mm_P5.00mm	THT
D1	Diode: 1N4001	1	D_DO-41_SOD81_P10.16mm_Horizontal	THT
D2	Diode: 1N4001	1	D_DO-41_SOD81_P10.16mm_Horizontal	THT
D3	Diode: 1N4001	1	D_DO-41_SOD81_P10.16mm_Horizontal	THT
J1	MPX5100DP	1	PinHeader_1×03_P2.54mm_Vertical	THT
J2	MPX5100DP	1	PinHeader_1×03_P2.54mm_Vertical	THT
J3	Connector 01×02	1	PinHeader_1×02_P2.54mm_Vertical	THT
J4	Micro SD	1	PinHeader_1×06_P2.54mm_Vertical	THT
J5	DS18B20	1	PinHeader_1×03_P2.54mm_Vertical	THT
J6	Connector 01×02	1	PinHeader_1×02_P2.54mm_Vertical	THT
J7	Connector 01×02	1	PinHeader_1×02_P2.54mm_Vertical	THT
J8	Connector 01×02	1	PinHeader_1×02_P2.54mm_Vertical	THT
J9	Connector 01×02	1	PinHeader_1×02_P2.54mm_Vertical	THT
L1	Inductor: 1 μH	1	R_Axial_DIN0207_L6.3mm_D2.5mm_P10.16mm_H	THT
R1	Resistor: 4k7 Ω	1	R_Axial_DIN0207_L6.3mm_D2.5mm_P10.16mm_H	THT
R2	Resistor: 4k7 Ω	1	R_Axial_DIN0207_L6.3mm_D2.5mm_P10.16mm_H	THT
R3	Resistor: 4k7 Ω	1	R_Axial_DIN0207_L6.3mm_D2.5mm_P10.16mm_H	THT
R4	Resistor: 10 kΩ	1	R_Axial_DIN0207_L6.3mm_D2.5mm_P10.16mm_H	THT
R5	Resistor: 1 MΩ	1	R_Axial_DIN0207_L6.3mm_D2.5mm_P10.16mm_H	THT
R6	Resistor: 10 kΩ	1	R_Axial_DIN0207_L6.3mm_D2.5mm_P10.16mm_H	THT
R7	Resistor: 1 MΩ	1	R_Axial_DIN0207_L6.3mm_D2.5mm_P10.16mm_H	THT
R8	Resistor: 10 kΩ	1	R_Axial_DIN0207_L6.3mm_D2.5mm_P10.16mm_H	THT
R9	Resistor: 1 MΩ	1	R_Axial_DIN0207_L6.3mm_D2.5mm_P10.16mm_H	THT
R10	Resistor: 1 MΩ	1	R_Axial_DIN0207_L6.3mm_D2.5mm_P10.16mm_H	THT
R11	Resistor: 22 Ω	1	R_Axial_DIN0207_L6.3mm_D2.5mm_P10.16mm_H	THT
U1	ESP32SIM800L	1	TTGO-TCALL-SIM800:TTGO-TCALL-SIM800	THT
U2	Inverter: 74HCU04	1	Package_DIP:DIP-14_W7.62mm	THT
Y1	Crystal 60 MHz	1	Crystal:Resonator-2Pin_W10.0mm_H5.0mm	THT

* THT: Through-Hole Technology mounting.

**Comparisons between SHYPROM and SHYPROM60**

This section highlights the main differences between the SHYPROM and SHYPROM60 sensors (Table A2).

**Table A2:** Main differences between SHYPROM and SHYPROM60 sensors.

Parameter	SHYPROM	SHYPROM-60	Variation
Operating temperature range (°C)	20–30	20–30	Same range
Operating frequency	600 kHz	60 MHz	Higher operating frequency
PCB structure	4-layer PCB	2-layer PCB	Simplified design and lower cost
PCB dimensions (mm)	95 × 100	95 × 100	Same dimension
Estimated cost (USD/unit)	~75	~65	Slight cost reduction
Transmission technology	GSM	GSM	Same technology
Sensor calibration/validation	1 soil	3 soils	Increased accuracy
