# Supplementary material for: Recent Technological Upgrades to the SHYPROM IoT-Based System for Monitoring Soil Water Status
Source: Sensors (Basel). 2025 Aug 9;25(16):4934. doi: 10.3390/s25164934 (PMC12390053; doi:10.3390/s25164934)
Supplement: Supplementary file 1 [file sensors-25-04934-s001.zip › 03 3D Printing Materials/How to assemble SHYPROM Sensor.pdf]

Users Guide for assembling the SHYPROM60 monitoring system

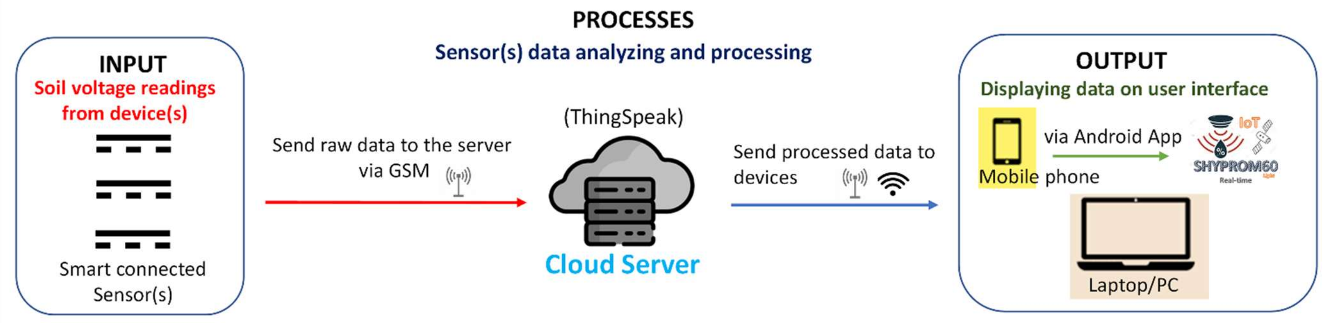

by Prof. *Alessandro COMEGNA*

Department of Agricultural, Forest, Food and Environmental Sciences (DAFE), University of Basilicata,

Potenza, Italy alessandro.comegna@unibas.it

Version 1.0 (*Spring 2025*)

This brief guide provides details for assembling the SHYPROM60 apparatus. Additionally, it provides details on the 3D-printed components necessary for assembling the monitoring system.

1 The Source Code Used to Program the Monitoring System

The source code which governs the hardware of the proposed monitoring system was written in C^{++} using the Arduino Integrated Development Environment (IDE). The architecture of this monitoring system is illustrated in the flowchart of Figure 1. Initially, the microcontroller initiates the general setup, which involves exiting sleep mode, initializing the SD card module and activating the internal modem. At this stage, the algorithm conducts a battery check before commencing the readings from various sensors: (i) soil temperature, (ii) soil voltage from the capacitive module at three distinct depths, and (iii) voltage output from tensiometers at two selected depths. Once obtained, the data are stored on the SD card and transmitted to the Cloud Server. Subsequently, the sensor enters sleep mode to minimize power consumption until the designated time slot concludes.

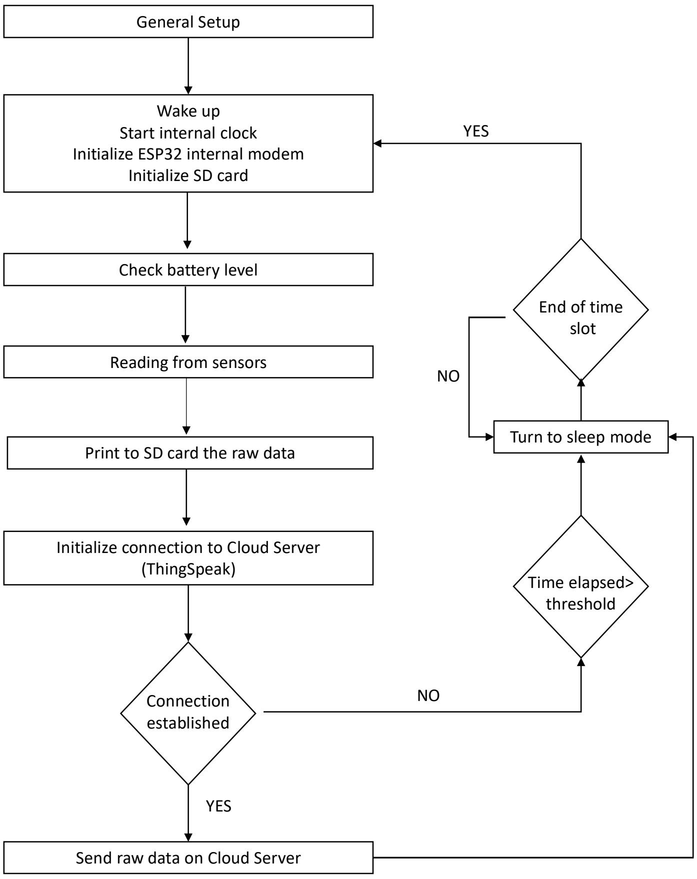

Figure 1. Flowchart of the IoT-based monitoring system.

2 SHYPROM60 assembling

The SHYPROM60 monitoring system also requires materials for the two probes that serve as tensiometers and for installing the electrodes of the capacitive-based module. This part of the sensor can be constructed using: i) plexiglass tubes with an outer diameter of 20 mm and ii) 3 cm long cylindrical tubes that act as electrodes (refer to local or online markets for sourcing these materials). To simplify the assembly of the capacitive module, consider 3D printing PLA cylindrical elements (see Probe Ring Bottom PLA.stl and Probe Ring PLA.stl files in the SHYPROM_RINGS folder). These PLA elements were designed for correctly wiring the electrical cables and securing the electrodes along the plexiglass tubes.

Once the SHYPROM60 monitoring system is assembled, the next step is to load the firmware file into the ESP32SIM800L control unit (refer to SHYPROM60.ino file, available for download from the SHYPROM60 Firmware folder). Before loading the firmware, it is crucial to set your Cloud URL (row 32 of the SHYPROM.ino file) and your Write API Key value (row 36 of the SHYPROM60.ino file); these parameters depend on the chosen Cloud platform. The Firmware SHYPROM60.ino file can be managed using the Arduino IDE (https://www.arduino.cc/en/software). Finally, the SHYPROM60 available data can be also visualized on the SHYPROM60_PRO Android app. To install the app on your device, you need the .apk file, which can be generated from the SHYPROM60_PRO.aia file (available for download from the SHYPROM60 Firmware folder). The latter file contains the source code and can be directly opened and modified on the MIT APP IN-VENTOR online IDE (https://appinventor.mit.edu/). The code must be rearranged for your specific requirements (i.e., it is necessary to modify the requested home URLs according to your Cloud specifications). Once modified, the MIT APP IN-VENTOR IDE creates the .apk file, which is the source file required for installation on Android-based mobile devices.

3 SHYPROM60 3D components

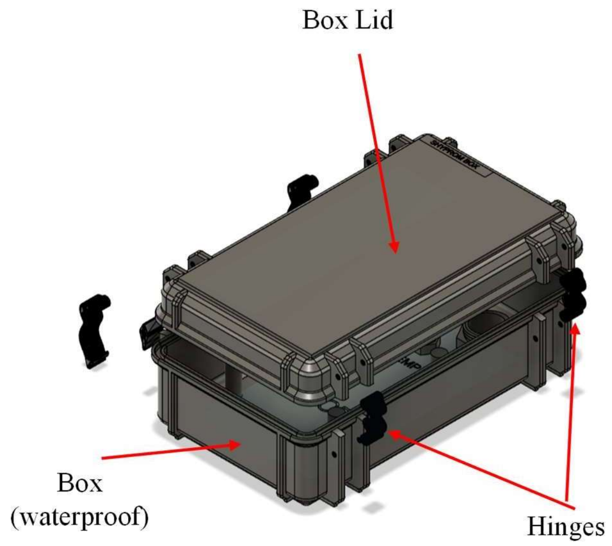

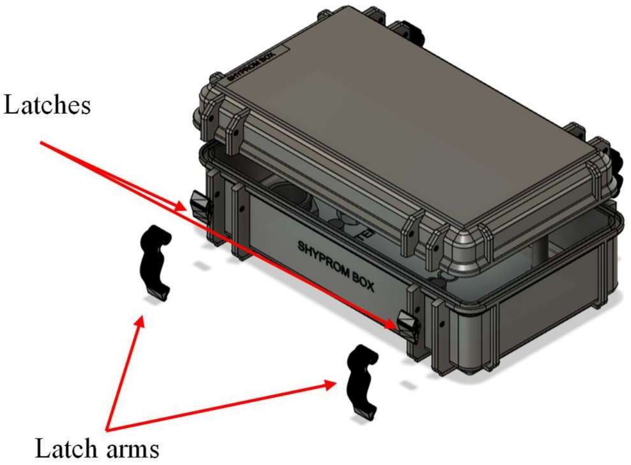

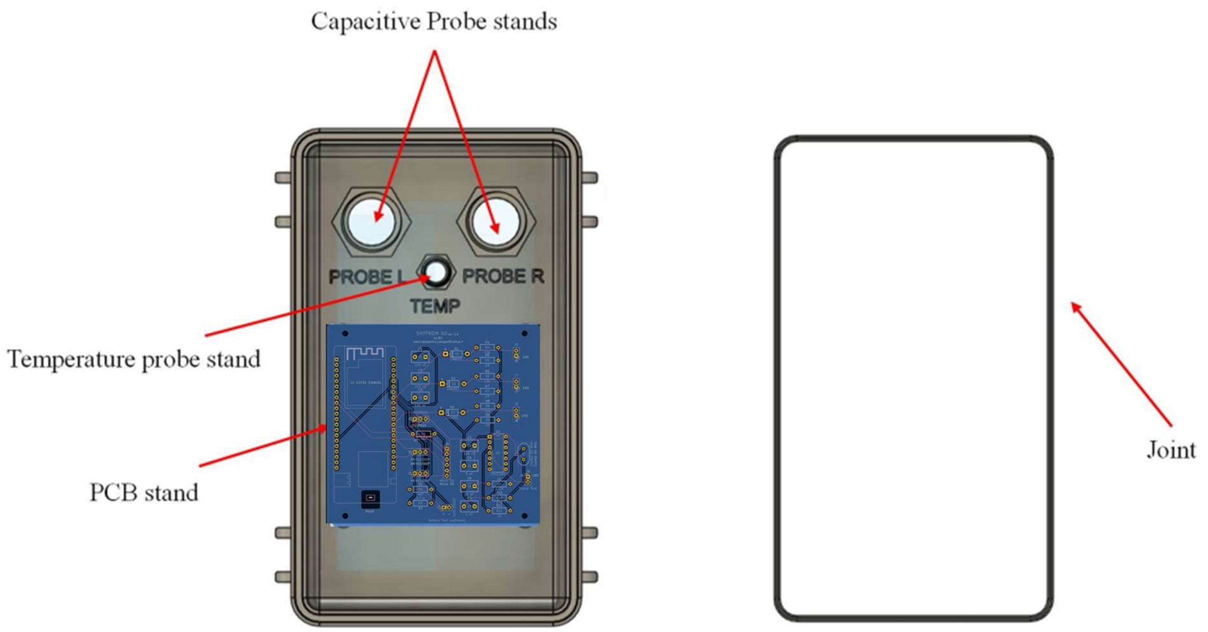

4. Example of electrode assembling (positioning on the plexiglass tubes)

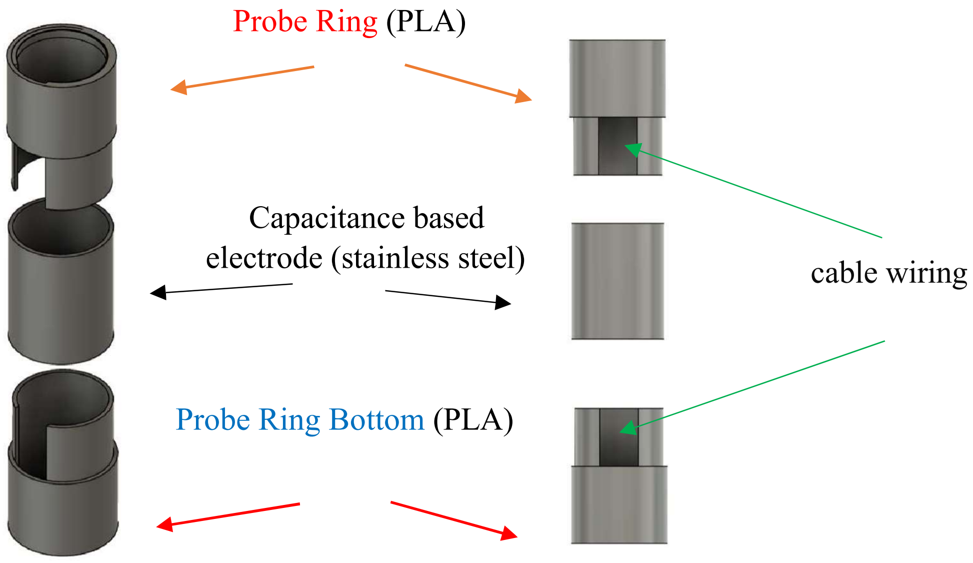

5 PCB Board with all the electronic components

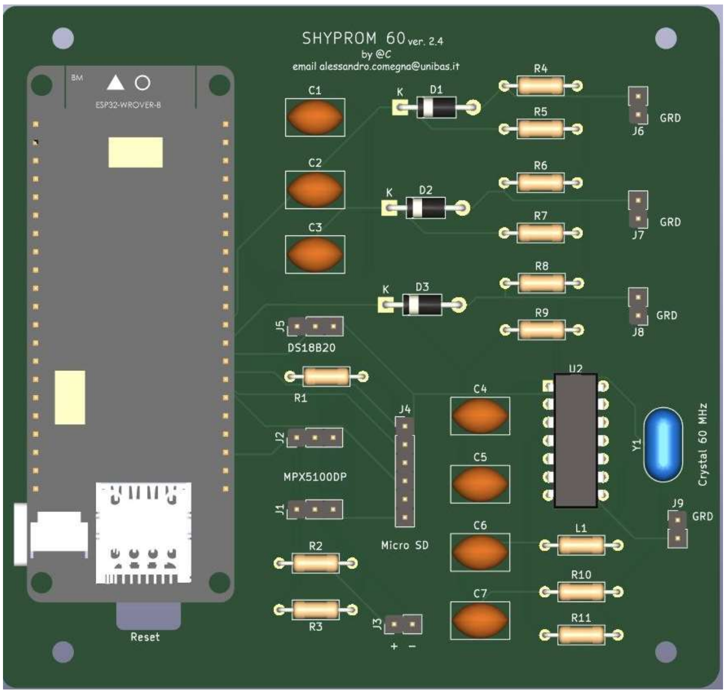

FRONT

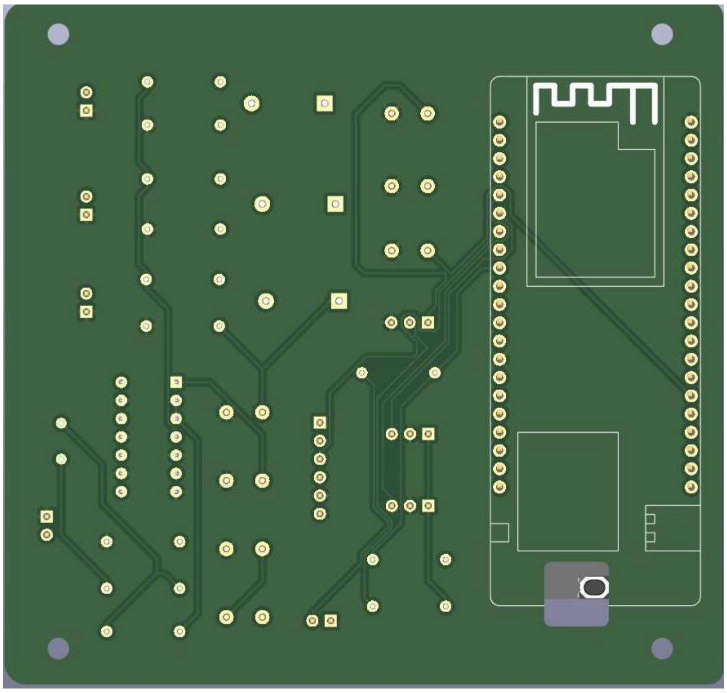

BACK

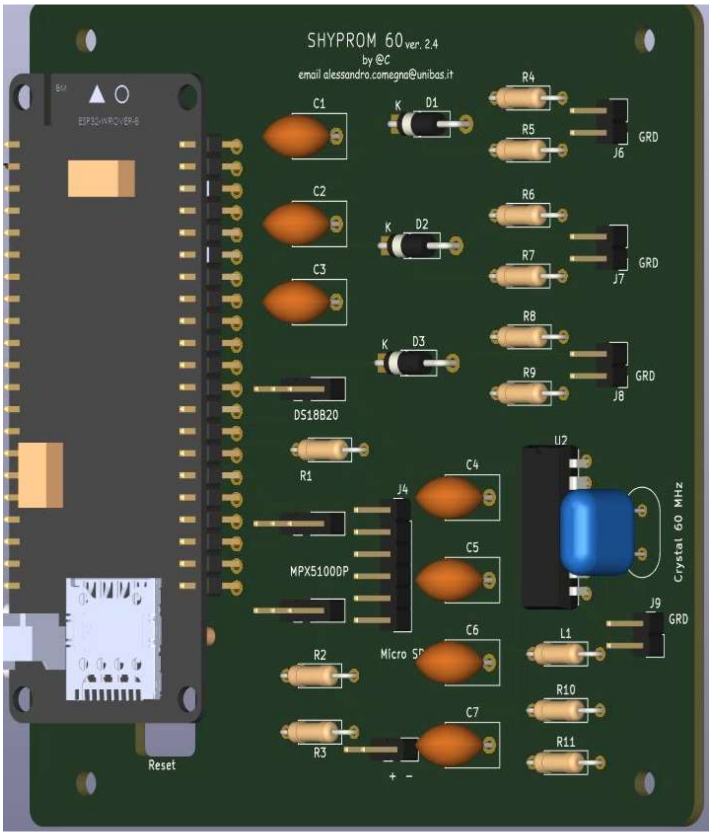

3D VIEW

6 Details on production costs

For implementing the electronic part of the SHYPROM monitoring system, two options are possible. The first option is to construct the board by yourself, following the electrical schematic. The estimated cost of fabricating one piece, including materials, is approximately USD 15-20 for the ESP32SIM800L board, USD 50-55 for two MPX5100DP pressure transducers, and around USD 2 for the other required components (listed in the Table A1).

For field applications, it is advisable to utilize a specialized electronics manufacturing service capable of producing low-cost printed circuit boards (PCBs). This option requires the Gerber and BoM files (refer to the Board Fabrication Files folder in the Supplementary Material). In this case, fabrication costs are around USD 5 for printing ten PCBs, excluding shipping.

7 Installing the SHYPROM Firmware

The firmware can be opened using the Arduino IDE, and in order to upload it to the ESP32SIM800L board, you must select **DOIT ESP32 DEVKIT V1** as the board.

By default, the Arduino IDE does not natively include boards from the ESP32 family. To upload a sketch to an ESP32 in the Arduino IDE, you first need to install the ESP32 board package.

Then, select the correct ESP32 board and the appropriate COM port from the Tools menu in the IDE. Finally, upload the sketch by clicking the Upload button.

7.1 Installing the ESP32 board package

Open the Arduino IDE. In the Boards Manager window, type ESP32 in the search bar. Click the Install button to start the installation process. This will download and install the ESP32 board.

Before loading the firmware, it is crucial to set your SIM data credentials (rows 31 and 34 of the SHYPROM60.ino file) and the Cloud URL (row 38 of the SHYPROM60.ino file) and yourWrite API Key value (row 42 of the SHYPROM.ino file); these parameters depend on the chosen Cloud platform.

The Firmware SHYPROM60.ino file can be managed using the Arduino IDE, (https://www.arduino.cc/en/software).

7.2 Installing the Android App SHYPROM60_PRO on your mobile device

Finally, the SHYPROM60 available data can be also visualized on the **SHYPROM60_PRO** Android app. To install the app on your device, you need the .apk file, which can be generated from the SHYPROM_APP.aia file (available for download from the SHYPROM60 Android App folder). The latter file contains the source code and can be directly opened and modified on the MIT APP INVENTOR online IDE (https://appinventor.mit.edu/). The code must be rearranged for your specific requirements (i.e., it is necessary to modify the requested home URLs according to your Cloud specifications). Once modified, the MIT APP INVENTOR IDE creates the .apk file, which is the source file required for installation on Android-based mobile devices.
